# Emerging Evidence for the Widespread Role of Glutamatergic Dysfunction in Neuropsychiatric Diseases

**DOI:** 10.3390/nu14050917

**Published:** 2022-02-22

**Authors:** Thomas McGrath, Richard Baskerville, Marcelo Rogero, Linda Castell

**Affiliations:** 1 Green Templeton College, University of Oxford, Oxford OX2 6HG, UK; thomas.mcgrath@gtc.ox.ac.uk (T.M.); lindy.castell@gtc.ox.ac.uk (L.C.); 2 Faculty of Health and Life Sciences, Oxford Brookes University, Oxford OX3 0BP, UK; 3 School of Public Health, University of Sao Paulo, Sao Paulo 01246-904, Brazil; mmrogero@usp.br

**Keywords:** glutamate, glutamine, excitotoxicity, inflammation, depression, neurodegeneration, neuropsychiatric conditions

## Abstract

The monoamine model of depression has long formed the basis of drug development but fails to explain treatment resistance or associations with stress or inflammation. Recent animal research, clinical trials of ketamine (a glutamate receptor antagonist), neuroimaging research, and microbiome studies provide increasing evidence of glutamatergic dysfunction in depression and other disorders. Glutamatergic involvement across diverse neuropathologies including psychoses, neurodevelopmental, neurodegenerative conditions, and brain injury forms the rationale for this review. Glutamate is the brain’s principal excitatory neurotransmitter (NT), a metabolic and synthesis substrate, and an immune mediator. These overlapping roles and multiple glutamate NT receptor types complicate research into glutamate neurotransmission. The glutamate microcircuit comprises excitatory glutamatergic neurons, astrocytes controlling synaptic space levels, through glutamate reuptake, and inhibitory GABA interneurons. Astroglia generate and respond to inflammatory mediators. Glutamatergic microcircuits also act at the brain/body interface via the microbiome, kynurenine pathway, and hypothalamus–pituitary–adrenal axis. Disruption of excitatory/inhibitory homeostasis causing neuro-excitotoxicity, with neuronal impairment, causes depression and cognition symptoms via limbic and prefrontal regions, respectively. Persistent dysfunction reduces neuronal plasticity and growth causing neuronal death and tissue atrophy in neurodegenerative diseases. A conceptual overview of brain glutamatergic activity and peripheral interfacing is presented, including the common mechanisms that diverse diseases share when glutamate homeostasis is disrupted.

## 1. Introduction

Mental disorders are one of the main causes of disability worldwide. In a pre-COVID pandemic WHO report of mental illness, 264 million people are affected by depression, 45 million by bipolar disorder, 20 million by schizophrenia, and 50 million by dementia [1].

Although improvements in medical care and disease understanding have reduced the symptoms and complications of mental illness, many conditions such as major depressive disorder (MDD), eating disorders, autism spectrum disorders (ASD), and neurodegenerative disorders remain challenging to control or reverse. The rate of development of treatment innovations in many neurological and psychiatric conditions has stagnated in recent years, indicating the need for paradigm shifts in approaches [2].

Since the 2000s, an increasing body of clinical research has demonstrated the involvement of glutamate neurotransmission in primary mood disorders and schizophrenia [3,4,5]. Glutamate is the most-abundant amino acid in the brain and was previously considered a fuel and synthesis substrate and immune mediator [6]. It was not until the 1980s that preclinical evidence emerged of glutamate as the main excitatory neurotransmitter in the central nervous system (CNS) with important roles in synaptic plasticity, learning, cognition, and memory [7,8,9,10].

There are several reasons for glutamate research lagging behind the research of other neurotransmitters. Varieties of glutamate receptors are more numerous and complex than the receptors of other neurotransmitters, making the development of ligands to bind and study receptor activity in vivo more challenging [11]. In addition, plasma glutamate cannot be used as a proxy measure of central activity, as the blood–brain barrier (BBB) functions to prevent excitotoxic levels of plasma glutamate from contacting brain tissue [12]. The third challenge in glutamate research has been differentiating glutamate from its inactive form glutamine by standard strength magnetic resonance spectroscopy (MRS). Elucidating the fundamental features of glutamatergic transmission has been facilitated by the development of an array of new tools, used to determine new receptors, regions of activity, and correlating pathology with clinical features. These methodological advances included highly sensitive analytical techniques, genetic advances, and improved radio-ligand imaging techniques [13,14]. The development of high-magnetic-field-strength scanning for MRS enables glutamate and glutamine distinction, and positron emission tomography (PET) allows localisation within precise areas of the brain [15,16]. Lastly, the glutamine/glutamate metabolic cycle within astrocytes enables glutamate to switch rapidly between roles as a neurotransmitter, immune mediator, or fuel substrate, making causal hypothesis testing very challenging. It is also possible that glutamate neurotransmitter research may have also been delayed by the focus on serotonin, dopamine, and other monoamine neurotransmitters over the decades. 

This review describes the current understanding of the mechanisms and functions of glutamatergic neurotransmission and metabolism, in health and disease. Mechanisms of the exacerbating effects of stress, inflammation, and other factors are discussed. There is a detailed focus on the role of glutamate in depression as this area is the most closely studied to date and serves to illustrate general themes in glutamate function and dysfunction. Emerging evidence for the roles of glutamate in other neuro-psychiatric conditions is then discussed.

The Diagnostic and Statistical Manual-V (DSM-V) classifies mental disorders into classes as follows: Neurodevelopmental disorders; schizophrenia spectrum and other psychiatric disorders; bipolar and related disorders; depressive disorders; anxiety disorders; obsessive-compulsive and related disorders; trauma- and stressor-related disorders; and neurocognitive disorders [17]. The diversity of neuropsychiatric conditions shown to be linked to glutamate testifies to its importance in virtually all areas of brain functioning. Glutamine and glutamate are both five-carbon amino acids that are biochemically interchangeable and therefore tend to share roles in maintenance and cell function. In the CNS, glutamate is a neurotransmitter and predominates in importance. In the periphery, glutamine actions tend to predominate in importance.

### 1.1. Glutamine/Glutamate in the Peripheral Circulation

Glutamine is the most abundant free amino acid in human muscle and plasma and is also found at relatively high concentrations in many human tissues. It is synthesized, stored, and released predominantly from skeletal muscle, for example, during strenuous exercise. Glutamine has versatile roles in cellular metabolism, immunity, nitrogen and pH homeostasis, and intermediary metabolism [18,19]. Glutamine has biosynthetic roles providing carbon and nitrogen for nucleotide synthesis (purines, pyrimidines, and amino sugars), nicotinamide adenine dinucleotide phosphate (NADPH), antioxidants, and many other biosynthetic pathways involved in the maintenance of cellular integrity and function [20]. Cells such as lymphocytes, neutrophils, and macrophages use glutamine as a fuel substrate under conditions of catabolic stress such as sepsis, recovery from burns or surgery, and malnutrition. Under these conditions, particularly in clinical studies, glutamine supplementation has been seen to augment T-cell function and reduce circulating inflammatory mediators, such as the cytokine IL-8 [21]. Several studies have demonstrated that the restoration of the plasma glutamine concentration results in partial attenuation of inflammation. Glutamine supplementation has not been shown to have any ergogenic effect in exercise studies [22]. The mechanism of the anti-inflammatory effects of glutamine is not well described; however, it has been suggested that regulation can be mediated by the balance of T-helper cell types 1 and 2 in the expression of pro-inflammatory and anti-inflammatory cytokines [23,24].

### 1.2. Glutamate Compartments in the Central Nervous System

Glutamate has long been known to be abundant in the brain, up to 15 mmol/kg [25]. From the 1930s, glutamate was known to have a central metabolic role and brain cells were understood to have high glutamate uptake activity, but research techniques were unable to discern detailed functioning in vivo. Although the neurotransmitter function of glutamate was suspected in the 1950s, this was not confirmed until the 1980s by radiolabelling techniques [26]. Glutamate is the predominant excitatory neurotransmitter in the CNS accounting for over 90% of excitatory function [27]. Glutamatergic synapses are found throughout the CNS but are especially concentrated in the hippocampus, caudate nucleus, thalamic nuclei, and the cerebellum [28].

Synaptic excitation occurs at micromolar concentrations, whilst intracellular glutamate and plasma glutamate levels are millimolar, and therefore strict compartmentalisation is important to prevent spillage and excitotoxicity. Glutamate cannot be enzymatically deactivated in the synaptic space after neurotransmission; therefore, astrocytes have an elaborate system of reuptake transporters to remove glutamate and maintain the space at micromolar concentrations. To control the combined brain load of glutamate and glutamine, the blood–brain barrier (BBB) of the CNS is designed to allow efflux and prevent influx [29]. The BBB protects most areas of the CNS from the much higher glutamate levels in the circulation (50 µM compared to 200 µM) [30]. The majority of glutamate in the brain is synthesized de novo by astrocytes, potentially leading to a gradual accumulation of brain glutamine/glutamate. Without the BBB efflux pathway, glutamate levels would progressively rise in the brain causing astrocyte swelling and cerebral oedema. Instead, glutamine is partially metabolized to NH4^+^ and glutamate by the BBB endothelial cells, allowing excess CNS glutamine and ammonia to diffuse out into the circulation [31]. This process enables the regulation of both the brain nitrogen balance and CNS glutamine. The BBB both permits glutamine efflux and prevents influx. Peripherally, the body produces 1000 mmol per day of ammonia, predominantly from muscle and liver metabolism, which the BBB prevents from entering the CNS in the form of ammonia or glutamine. However, at higher levels, for example, in liver disease, the BBB becomes breached and excess glutamate causes excitotoxicity. The neurotoxic effects of excess circulatory ammonia or glutamate are known as hyperammonaemic encephalopathy. Commonly resulting from acute or chronic liver disease or adverse drug side effects, this condition features psychomotor, intellectual, and cognitive abnormalities with emotional/affective and behavioural disturbances and, as such, this condition provides inadvertent clinical evidence towards the glutamate hypothesis of the effects of excess glutamate on the CNS [32].

Lastly, the excitatory nature of glutamate neurotransmission and the complex systems needed to maintain concentration gradients is extremely energetically expensive, accounting for 80% of entire brain energy expenditure, forming 20% of the total body energy expenditure, and revealing the reason prolonged mental concentration, involving widespread glutamatergic activity, causes sensations of physical fatigue [33,34,35]. Whether it is by luck or design, glutamate is also an excellent oxidative substrate and, through the glutamine–glutamate cycle and mitochondrial malate–aspartate cycle, enables glutamatergic neurotransmission to effectively provide its own local power supply, thus avoiding potential energy mismatch issues [36].

However, the high metabolic demands of glutaminergic neurotransmission in the brain also entail a correspondingly high oxygen demand. The clinical consequence of this is the brain’s extreme vulnerability to hypoxia, manifested in clinical conditions such as ischaemic stroke, cerebral palsy, and altitude sickness.

Notably, brain areas with high glutamatergic activity, e.g., the hippocampus and thalamus, are also seen to be the most sensitive to hypoxia [37].

### 1.3. Glutamatergic Receptors

The complex functions that glutamate serves in excitatory neurotransmission, but also in modulating and signalling between neurones and astroglial cells, is due to the wide range and complexity of glutamate receptors (GluRs). Two distinct classes of receptors are recognised on the basis of functional differences: Inotropic GluRs (iGluRs) are ligand-gated ion channels and metabotropic GluRs (mGluRs) are G protein-coupled receptors (GPCRs) that control cellular processes via G protein signalling cascades [38]. The first iGluR and mGluR members were cloned in 1989, and intense research into receptor subunits and subtypes and ligands has continued ever since [39]. Individual cells are seen to display combinations of subtypes of each of these classes, which glutamate activates indiscriminately. Thus, individual pre-synaptic glutamate releases can encode complex post-synaptic responses from receptors acting in concert, both on neurones and microglial cells. Specific iGluR agonists have revealed the existence of receptors of α-amino-3-hydroxy-5-methyl-4-isoxazolepropionic acid (AMPARs), kainate (KARs), and *N*-methyl-D-aspartate (NMDA receptors) as the three main subfamilies [40]. The expression pattern of different iGluR subtypes varies in different brain regions, but their expression is widespread, and individual cells typically express multiple different iGluR subtypes [41]. The eight mGluRs are divided into groups I, II, or III subfamilies, based on sequence homologies and their preferred Gα protein signalling partners. The molecular diversity of both iGluRs and mGluRs is increased by a number of mechanisms including the formation of heteromers within each subfamily [42]. Post-receptor proteins are also subject to post-translational modifications, and some iGluRs have auxiliary subunits that modulate functions. In summary, there is a vast array of possible receptor combinations at the cell surface encoding numerous signal types. 

### 1.4. Astrocyte Control of Glutamate Levels

**Figure 1:**
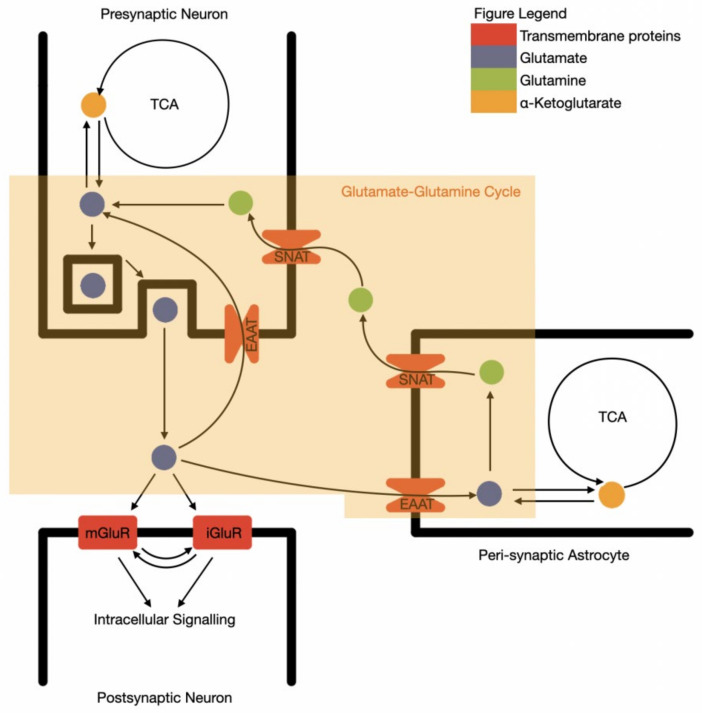
The synaptic functional unit of glutamate neurotransmission and recycling. TCA—tricarboxylic acid cycle; SNAT—sodium-coupled amino acid transporter; EAAT—excitatory amino acid transporter; mGluR—metabotropic glutamine receptor; iGluR—ionotropic glutamine receptor.

Due to the excitatory and potentially toxic nature of glutamate, the precise control of synaptic space glutamate levels by surrounding astrocytes is essential. Glutamate is extracted from the synaptic space by astrocytes through glutamate transporters: GLT-1, GLAST, and EAAT3,4,5, converted into non-excitatory glutamine and then recycled back to the neurone in a process known as the glutamate–glutamine cycle (Figure 1) [43]. This is known as an “open cycle”, as glutamate alternatively acts as a cellular energy substrate, via conversion to alpha-ketoglutarate and entering the TCA cycle [44]. The oxidised glutamate is then replaced with similar rates of de novo glutamate synthesis from glucose, via pyruvate and alpha ketoglutarate in astrocytes.

An additional role of glutamate in astrocytes is to facilitate the entry of the reduced form of NADH from the cytosol of astrocytes into the mitochondrion, to enable glycolysis to proceed (the malate–aspartate shuttle). Astrocytic metabolism is therefore strongly influenced by glutamate and, conversely, astrocytes have a primary role in regulating glutamate neurotransmission. This close relationship therefore closely binds glutamatergic neurotransmission with cellular energy metabolism, although the precise mechanisms are not fully understood. Loss of glutamate compartmental homeostasis therefore has bifold consequences leading to deficient or excess synaptic glutamate transmission and brain energy dysfunction with inflammation, leading to neurodegeneration. 

Astrocytes therefore form part of a functional unit with the neurone, rather than simply providing neuronal support as previously thought. The most studied clinical area of CNS glutamatergic dysfunction is in depression, where many domains of brain functioning are affected and where intense research into glutamate receptor subtypes and therapeutic modulating agents is continuing. 

As a result of this, depression research has revealed new facets of glutamate neurophysiology, in particular with relation to stress and inflammation. In this review, therefore, depression is described in detail, and it is proposed that similar mechanisms underlie a range of other neuropsychiatric conditions.

## 2. Depression

Mental disorders are among the leading causes of the global health-related burden. The Global Burden of Diseases, Injuries, and Risk Factors Study (GBD) 2019 showed that the two most disabling mental disorders were depression and anxiety disorders, both ranked among the top 25 leading causes of the health-related burden worldwide in 2019 [1]. This burden was high across the entire lifespan, for both sexes, with global distribution and rising incidence. The total estimated number of people living with depression worldwide increased by 18.4% between 2005 and 2015 to 322 million equating to 4.4% of the world’s population, with a lifetime risk of 15–18%. In terms of disability, in 2017, 163,044,100 cases of Major Depressive Disorder (MDD) were counted, ranking third in the causes of years lived with disabilities [45]. Perhaps more importantly, no reduction in the global prevalence or burden has been detected for depression or anxiety since 1990, despite compelling evidence of interventions that reduce their impact [46].

Depression is defined by diagnostic criteria from the Diagnostic and Statistical Manual 5 (DSM5). Essential symptoms should include either depressed mood or anhedonia (loss of interest or pleasure), with additional symptoms including appetite or weight changes, difficulty sleeping, diminished ability to think or concentrate, fatigue or loss of energy, feelings of worthlessness, or excessive guilt and suicidality [46]. The heterogeneity of the clinical presentation, presence of comorbid conditions, and psychosocial confounding variables is challenging for the reproducibility of studies of the underlying mechanisms. In addition, the inaccessibility of the human brain for direct observation and measurement has led to indirect animal modelling, theory-led understanding, and empirical treatment approaches. 

MDD is characterized by the impairment of monoaminergic and neurotrophin mechanisms, changes in mood due to reduced levels of serotonin and dopamine, and impaired brain health by reduced neurotrophin levels. The prevailing treatment paradigm of depression since the 1960s has centered around serotoninergic, noradrenergic, and dopaminergic activity, because experimentally, these monoamines have been linked to mood, cognitive functions, sleep regulation, and stimulation of the reward center. Empirically, drugs increasing synaptic monoamine levels, such as uptake inhibitors and enzyme inhibitors, are effective in reducing clinical symptoms and therefore have formed the rationale for the “Monoamine theory of depression” [47].

Unfortunately, the monoamine model poorly accounts for the 30% of people with MDD who do not achieve remission despite multiple treatments, or who develop suicidal behaviour. This treatment-resistant depression (TRD) is extremely challenging to manage clinically and is compounded by the stagnation of drug innovation in recent decades [48]. The model also provides little mechanistic explanation of the strong connections of MDD with stress or inflammation or the benefits of non-drug interventions such as physical activity on the CNS.

### 2.1. The Glutamate Hypothesis of Depression

The glutamate hypothesis of depression was proposed in the 1990s, when antagonists of the *N*-methyl-D-aspartate (NMDA) receptor, an ionotropic glutamate receptor, were found to possess antidepressant-like mechanisms of action in mice [49]. In a seminal human trial by Berman in 2000, ketamine (a long-standing anaesthetic agent and NMDA receptor antagonist) demonstrated rapid-onset mood improvement in MDD, including in cases of treatment-resistant depression (TRD) [4]. 

Current hypotheses of glutaminergic sites of action of ketamine are via NMDA receptor inhibition at the synapse and on GABA interneurones and through AMPA receptor activation. Ketamine antagonises the NR2B subunit of NMDA receptors on GABA neurones, and the resulting block on inhibition causes glutamate excitation to increase [50]. The immediate antidepressant effects appear to be linked to longer-term downstream mechanisms regulating synaptic plasticity, including the brain-derived neurotrophic factor (BDNF), eukaryotic elongation factor 2 (eEF2), and the mechanistic target of rapamycin (mTOR) expression in the hippocampus and prefrontal cortex [51]. Ketamine also acts on other glutamate pathways, so the precise mechanism of overall glutamatergic system suppression is still unclear. In addition, ketamine inhibits noradrenaline, dopamine, acetylcholine, and serotonin receptors, making precise attributions of causality difficult. Certainly specifically developed pure glutamate antagonists exert less clinical effect than ketamine [3]. Similar to other rapidly acting antidepressants, the ketamine-induced, BDNF-mediated increase in synaptic plasticity and structural changes accounts for the observed longer-term clinical benefits and the greater adaptive ability to stressful environments. In recent research, excitotoxicity damage was induced on astrocytes in the anterior cingulate cortex (ACC) of mice [52]. Although the glial damage was focal, the effects of reduced BDNF and reduced serotonin were detectable as a global change in both hemispheres, indicating the role of ascending and descending projections in amplifying local glutamatergic damage and perhaps explaining the varied clinical symptoms of depression.

### 2.2. Glutamatergic Imaging Studies 

Magnetic resonance spectroscopy ((1)H-MRS) enables the detection of variations of glutamine and glutamate concentrations across brain regions. Previous studies have suggested that glutamate levels are decreased in anterior brain regions. The heterogeneity of clinical cases in depression, treatment status, and low magnetic field strength produces inconsistency in results. However, a meta-analysis by Luykx detected a significant increase in glutamate in the anterior cingulate cortex in people with MDD compared to healthy controls [53].

This was concordant with a 2019 meta-analysis of 49 studies. The study also showed a decrease in glutamate in this region in successfully treated patients, adding evidence to excess glutamate as the cause of symptoms [54]. However, a high field strength (7T) MRS study found no difference in anterior cortical glutamate levels between cases of MDD and healthy controls, although increased glutamatergic activity was detected in the pathways to the basal ganglia [55]. A recent MRS study of depression found that increased total glutamate/glutamine (Glx) corresponded to more severe or untreated MDD and was correlated with reductions in GABA levels [56]. This suggests that impaired GABA functioning plays a role in glutamate excitotoxicity-induced depression. Despite this, a 2021 study found no deficit in GABA levels or receptor availability when combining MRS and flumazenil-labeled PET to examine anterior cortical and whole-brain GABA activity in MDD and controls [57]. In conclusion, despite significant imaging improvements and intense research over 20 years, the precise interactions of glutamatergic systems with other neurotransmitters in depression remain incompletely understood. 

### 2.3. Glutamate Mediates the Relationship of Stress with Depression

Intuitively, stress is closely linked to depression because a low mood is often associated with the unpleasant nature of stressors. However, stress and depression also share physical biochemical connections via glutamate neurotransmission, which mediates both mood function and stress responses in the brain. Acute stressful life events such as bereavement, divorce, financial or criminal issues, and illness produce widespread CNS responses such as anxiety, disturbed or excessive sleep, fatigue, memory and cognitive problems, in addition to depressed mood, and sometimes progressing to suicide [58]. 

Psychological stress, although incompletely understood, can be reproduced in experimental conditions, creating subtypes such as intermittent, restrained, or unpredictable stress, enabling biological correlates to be detected in animal models or human imaging. Acute stress induction is accompanied by well-documented increases in catecholamines, from sympathetic nervous stimulation (SNS), alongside corticotropin releasing factor (CRF), ACTH, and glucocorticoids from the hypothalamic–pituitary–adrenal (HPA) axis. Less well known and understood are the stress-induced rises in plasma IL-6, TNF-alpha, and monocyte nuclear-factor kappa-B (NFkB), despite the adjacent HPA axis normally exerting an immunosuppressive effect [59,60,61].

The mechanism may be partially actioned via the SNS, as chronic and repeated stress causes B-adrenoceptor-activated monocyte pro-inflammatory expression and monocyte migration to the CNS [62]. 

Stress from adverse events in early life further amplifies these pro-inflammatory responses to stress, possibly by shielding them from the increased levels of immunosuppressive glucocorticoids, although the mechanism is not fully understood [63].

The resulting chronic pro-inflammatory states may explain the connection between adverse life events in childhood and subsequent depression and chronic physical disease. Childhood Adverse Life Experiences (ALEs) are associated with a 2.4-times increase in mortality at the age of 65, compared to controls [64].

HPA axis activation is initiated when the corticotropin-releasing factor (CRF) is expressed by cells in the paraventricular nucleus (PVN) of the hypothalamus. These cells are triggered by glutamatergic excitation via glutamate receptors, demonstrating the close relationship between glutamatergic activity and the endocrine stress response [65].

Common to many glutaminergic microcircuits, GABA interneurones control the effect of excitation by inhibiting CRF cell secretion. GABA inhibition is itself deactivated when GABA ion channels open, causing depolarisation. Depolarisation is regulated by chloride cotransporters, which determine the transmembrane electrochemical gradient [66]. In animal models, psychological stress and the inflammatory mediator IL-6 affect the number and function of these cotransporters and therefore act to deactivate GABA inhibition, hence increasing CRF secretion [67]. Therefore, in the short term, stress-induced deactivation of GABA, and hence CRF and HPA activation, is an appropriate physiological response to a perceived threat.

However, prolonged stress and different forms of experimental stress cause continual suppression of GABA inhibition, leading to the hyperactivation of glutamatergic stimulation of CRF cells and exhaustion of the HPA axis with low levels of glucocorticoids. This is sometimes referred to as “the GABAergic deficit hypothesis of major depressive disorder” [68]. Low circulating glucocorticoids are also seen in atypical depression, PTSD, and suicide attempts [69].

CRF cell exhaustion and consequently reduced circulating glucocorticoids remove the suppressive influence the HPA axis exerts on inflammatory mediators; these then increase in both the circulation and CNS, causing dendritic damage and the transformation of astrocytes into pro-inflammatory phenotypes, described below. Inflammatory mediator neurone and astroglial damage are seen in prolonged or recurrent stress disorders and severe chronic depression. Although the underlying precipitants and perpetuating causes are not fully established, external agents that promote glutamatergic excitation and suppress GABA inhibition are clearly involved. 

Part of the perpetuation of HPA axis activation occurs as a result of a self-amplification effect, whereby HPA glucocorticoids provide feedback to the CNS and activate glucocorticoid receptors (CR) on the original glutamate presynaptic neurones, provoking further CRF cell activation. This likely serves a useful amplification function in appropriate stress responses to short-term stressors, but in persistent CR receptor activation, this causes neuronal exhaustion and apoptosis with macrostructural changes [70].

Another effect of the CR receptor-induced rise in extracellular glutamate is the astrocytic release of ATP causing microglial proliferation, the release of pro-inflammatory mediators, the release of BDNF, and the reduced GABA inhibition of glutaminergic activity [71]. 

In animal models, the effects of persistent glucocorticoids on glutamate neurones are increased glutaminergic excitability with astrocytic decline, reduced astroglial plasticity, and reduced dendritic connectivity in hippocampal and frontal cortex regions [72,73]. This is clinically detectable as depression of mood with eventual neurodegenerative structural changes that affect cognition.

Clearly, stress, anti-inflammatory HPA-stress responses, and pro-inflammatory mediators have a complicated and sometimes seemingly contradictory relationship, although this may simply reflect different dynamic phases in the physiological stress response and the differing requirements over time, similar to the catabolic/anabolic phases of repair following tissue damage. This physiological defence system, which evolved under ancestral conditions of short-term stresses, may become pathological under extreme or persistent psychological stress conditions. 

The notion that even brief depression of mood should be an inherent constituent of an ancestral protective system seems paradoxical. The evolutionary significance of this is unclear, although the energy savings from reduced glutamatergic activity and physical activity may be advantageous in certain situations of acute stress from infection. These energy-conservation symptoms are seen in the “sickness behaviour” of people with severe infections featuring behavioral changes, such as anorexia, fatigue, loss of interest in usual daily activities, social withdrawal, listlessness or malaise, hyperalgesia, sleep disturbances, and cognitive dysfunction [74]. Proponents of the “pathogen defence hypothesis of depression” cite human genomic studies supporting this association, indicating the ancestral and continued proximity of alleles for depression and pro-inflammatory factors [75].

### 2.4. Glutamate in Inflammation Related Depression 

Whilst CNS inflammatory mediators form part of the depression-associated stress response above, inflammation per se can provoke depression [76]. Equally, people with depression have increased plasma IL-6, indicating a probable bidirectional relationship. A meta-analysis of 24 studies in depressed patients found that individuals with major depression had significantly higher TNF-α and IL-6 plasma concentrations in comparison to controls [77].

Regardless of whether the origin is centrally derived or peripheral, inflammatory mediators produce similar effects on glutamate microcircuits to those seen with stress, namely glutamate excitotoxicity and atrophy with NMDA-stimulated astrocyte atrophy and microglial proliferation. IL-6, as a principal mediator of inflammation, derives peripherally from adipose tissue, muscle, and leukocytes and binds to IL-6 receptors (IL6R) on neutrophils, monocytes, and subsets of T-cells. IL-6 can also bind to soluble receptors (sIL6R) in plasma. sIL6R is produced by hepatocytes and neutrophils and are increased 2–3-fold in plasma during inflammation [78]. sIL6R is able to cross the BBB and cause IL-6 membrane-bound receptor stimulation in the CNS. IL-6 is also secreted endogenously in the CNS by microglia [79]. In the CNS, IL-6 can confer either neuroprotective effects via membrane-bound “classical” pathways or pathological effects via sIL6R [80]. A soluble form of IL-6R is an agonist capable of transmitting signals through interaction with the gp130 protein. In vivo, the IL-6/sIL-6R complex stimulates several types of target cells, which are unresponsive to IL-6 alone, as they do not express the membrane-bound IL-6R. This process is named trans-signalling. As a result, IL-6 can result in contradictory outcomes depending on stressor types and local cellular conditions. Activation of IL-6 receptors on microglia causes cellular transformation of the pro-inflammatory M1 phenotypes. This stimulates astrocytes, via the gp130 receptor, to transform into a “neurotoxic reactive A1” form. The A1 phenotype of astrocytes is toxic to some types of neurones, causing cell death and eventual cognitive deficits. This IL-6 activation of microglial cells is blocked by glucocorticoids acting on microglial CR receptors, showing a neuroprotective effect of the HPA axis in CNS inflammation. This is in contrast to the neurotoxic effect of glucocorticoids on glutamate neurone CR receptors in stress-related conditions described above [81].

In addition, inhibitory GABA interneurones are suppressed by IL-6 trans signalling, thereby increasing glutamatergic activity and causing anxiety and depressive symptoms. The enhanced glutamate signalling causes exaggerated HPA axis and sympathetic nervous system responses to stress during inflammatory states, which provoke somatic symptoms of anxiety such as tachycardia. 

### 2.5. The Kynurenine Pathway 

An important determinant of inflammation in CNS glutamatergic systems is the kynurenine pathway. The kynurenines are bioactive metabolites of tryptophan catabolism. Much interest has focused on members of this pathway in terms of mediating inflammatory CNS conditions. In the brain, the pathway divides into two branched pathways depending on local conditions: Either neurotoxic metabolites including quinolinic acid, QuinA (an NMDA receptor agonist), or neuroprotective metabolites including kynurenic acid, KynA (an NMDAr antagonist). Initially, the kynurenine pathway was thought to cause depression by depleting serotonin, but the principal site of action is now known to be glutamatergic transmission [82]. The ratio of KynA to QuinA has been demonstrated to be low (i.e., neurotoxic), driven by the excess QuinA released from microglial cells, in the anterior cingulate cortex when assessed in depression [83]. Moreover, the kynurenine pathway itself is modulated peripherally in adverse conditions such as infection and stress, or beneficially in exercise, by muscle enzyme increases or decreases causing changes in the KynA:QuinA ratio [84]. Exercise modulates the kynurenine pathway in muscle, producing myokine-like metabolites that promote an increase in the KynA/QuinA ratio. This is responsible for the beneficial effects of exercise such as improved energy homeostasis, the promotion of an anti-inflammatory environment, and neuroprotection, and is thought to be one of the mechanisms underlying the beneficial effects of exercise on depression [85]. 

Whilst exercise is unequivocally effective in reducing depression symptoms, other non-pharmacological glutaminergic-based treatments gaining evidence up to the systematic review level that may operate through this pathway are transcranial magnetic stimulation, electroconvulsive therapy, and deep brain stimulation [86,87,88].

### 2.6. Chronic Disease, Inflammation, and Depression

Although depressive behaviours, as part of inflammatory responses, may be evolutionarily advantageous in situations of acute infection, in chronic diseases, the link between depression and inflammation becomes counterproductive. There is increasing clinical evidence of chronic disease-associated depression (CDAD), where the systemic pro-inflammatory state is prolonged, excessive, or inappropriate, and circulating inflammatory mediators exert damage on neurones and astroglia. Chronic low-grade pro-inflammatory conditions underlie many chronic disease domains, including cardiovascular disease, metabolic diseases such as Type 2 diabetes, auto-immune diseases, such as rheumatoid arthritis, and chronic infections such as HIV. Disease-associated depression is therefore much more common than previously appreciated, comprising 20% of all patients with chronic disease [89]. Of people with Type 2 diabetes, 38.85% present with depression [90].

Depression as a non-intuitive but integral component of chronic disease is a recognised but under-addressed concept in routine healthcare [91]. This is possibly because low mood, overeating, and weight gain are mistaken as behavioural responses to the symptom burden, rather than being biochemically driven. In inflammatory bowel disease patients, increased circulating cytokines cause the co-occurrence of depression by the mechanisms stated above [92]. Similar correlations occur with other pro-inflammatory chronic diseases, including rheumatoid arthritis, cardiovascular disease, chronic obstructive pulmonary disease, and psoriasis [93,94,95,96]. Some non-disease pro-inflammatory states such as adiposity show similar associations with depression. In some diseases, the biochemical abnormalities of the disease itself impact glutamate-related depression. For example, in diabetes, recent research demonstrated, in vitro, that as little as 7 days of high glucose levels disrupt glutamate homeostasis through AMPA and NMDA upregulation and increased extracellular glutamate. This resulted in neurotoxicity and neuronal death [97]. Occasionally, in diseases such as inflammatory bowel diseases, immunomodulating treatment (anti-TNF) can inadvertently help reduce the inflammatory-mediated depression and has been shown to reduce symptoms even after adjusting for the anxiety associated with the condition [98]. In animal models of chronic disease-induced depression, hippocampal microglia secrete inflammatory mediators with upregulation of the GR/NF-κB signalling pathway. Interestingly, the underlying process and clinical symptoms are reversed by dexamethasone (a glucocorticoid receptor agonist), suggesting possible future treatment targets for this condition [99].

The importance of classifying disease-associated depression as a distinct clinical entity is to avoid a treatable clinical condition passing unrecognised during healthcare. Untreated depression impacts a person’s ability to self-manage the chronic condition and to initiate the lifestyle changes these conditions require, e.g., dietary changes, physical activity. Research into treatments is continuing, and studies indicate that conventional pharmaceutical and psychological treatment strategies are effective for disease-associated depression, if diagnosed promptly [100]. 

### 2.7. Chronic Disease-Induced Neurodegeneration

This review has described how glutamatergic excitotoxicity in primary and stress-related depression is seen to progress to neuronal atrophy, causing cognition and memory deficits. It seems reasonable, therefore, to suspect that the pro-inflammatory processes causing disease-induced depression also progress over time to cause neurodegeneration and irreversible effects on brain structure and function. Research suggests that this is the case across a range of diverse diseases, all of which feature pro-inflammatory states. There are links observed between inflammatory bowel disease and Parkinson’s disease, neurodegeneration resulting from chronic obstructive pulmonary disease, and cognitive deficits and arthritis [101,102,103]. Long-standing diabetes is associated with both Parkinson’s disease and dementia-like syndromes [104,105]. This further emphasises the need for awareness of CNS complications as part of chronic disease management.

There is a significant body of evidence for the neuroprotective effects of exercise in chronic disease states. Physical exercise moderates or reverses many of the processes of neuroimmune toxicity at the cellular and molecular levels by improving the production of neurotrophic factors, neurotransmitters, and hormones, and promoting synaptic plasticity, neurogenesis, angiogenesis, and autophagy [106]. This is discussed further in Section 12.

## 3. Glutamatergic Activity in Bipolar Disorder

Bipolar depression (BD) is a disease featuring recurrent depression and hypomania, whose onset is often during teenage years. Bipolar disorder confers the highest suicide rate of all mental illnesses [107]. The advent of proton MRS and PET has enabled detailed study of this group of conditions, whose pathophysiology remains incompletely understood. Disturbances in hippocampal glutamate levels and NMDA functioning have been implicated by neuroimaging studies [108]. Data also demonstrate that the lack of tight regulation of hippocampal NMDA functioning may affect the glutamate-mediated cognition required for memory and processing speed, which are essential for assessing the environment and sense of reality [109]. Despite the predominating presence of low mood states in bipolar disorder, the DSM-V has separated bipolar disorder from depressive disorders and placed it into a separate diagnostic category. This is in recognition of its bridge position in terms of risk factors and pathological findings—between psychotic and depressive disorders [110]. Bipolar disorders are characterised by the presence of a depressive episode preceded or succeeded by a hypomanic or manic episode (DSM-V), and they exhibit a lifetime prevalence of 2.4% in the global population [111]. 

Bipolar disorder features major depression components and can display episodes of psychosis similar to schizophrenia. Glutamatergic dysfunction is implicated in the pathogenesis of bipolar disorder, although notably, a Cochrane systematic review of the effects of ketamine and other glutamate modulators on symptoms failed to detect a significant effect in meta-analyses [112]. In animal models, aberrant synaptic plasticity is seen in the sites of dopamine and glutamate crosstalk and postsynaptic protein density (PSD) meshes [113]. Kynurenic acid blocks both dopamine action and glutamate, causing rises in extracellular levels, which appear to be related to psychotic symptoms and cognitive impairments [114]. This implies that inflammation is a factor, as the kynurenine pathway is critically regulated by cytokines. Both IL-6 and IL-1beta are seen to be elevated in bipolar disorder and stimulate kynurenic acid [115]. Notably, many of these changes also feature in schizophrenia, as discussed below.

## 4. The Glutamate Hypothesis of Schizophrenia

Schizophrenia is a severe neurodegenerative condition, featuring “positive” symptoms such as delusions and disordered thinking with loss of sense of reality, and “negative” symptoms such as blunted cognition, emotion, and social interaction [116]. The symptoms often appear spontaneously in the teenage years, and the aetiology is not understood. There is evidence of neurodevelopmental, genetic, and environmental influences in the pathogenesis of schizophrenia, although the direction and causality of the associations with glutamate function and inflammation, and the sequence of events, prior to psychosis, are still uncertain. Until the 1980s, treatment had targeted dopamine receptors and aetiological theories had centered on dopamine due to dopamine-associated positive symptoms [117]. As a result, dopamine-related antipsychotic medications reduced only positive symptoms. The “hypoglutamatergic hypothesis of schizophrenia” arose from the evidence of NMDA receptor antagonists such as ketamine reproducing schizophrenia-like symptoms, as well as evidence that agents reducing glutamatergic activity reduced both positive and negative schizophrenia symptoms [118]. In addition, a significant minority of patients taking dopamine-related agents did not respond to standard treatment at the time [119]. The dopamine and glutamate hypotheses are not mutually exclusive, however, as dopamine neurones interact closely with glutamate neurones. Similar to the findings of brain MRS studies examining glutamate in depression, studies in schizophrenia have also not historically been concordant [120]. Some meta-analyses have indicated significant declines in glutamate in the mPFC, whilst others have concluded non-significant glutamate changes in the same region [121,122]. Stratifying cases by medication status, medication response, and disease course, a review by Nakahara et al. found small-to-moderate glutamate activity reductions in the basal ganglia, hippocampus, and prefrontal cortex in symptom-controlled patients [123]. Reduction in NMDA receptor function causes increased glutamate levels and excitotoxicity. The original assumption was of generalised NMDA reduction throughout the CNS, but studies show that a reduction in the thalamus, in particular, reduces the glutamatergic stimulation of GABA inhibitory interneurones acting on excitatory tracts connected to main cortical areas [124]. This results in a widespread surge of excitatory glutamatergic activity. The NMDA blockage or reduction also leads to increased extracellular glutamate, which leads to AMPA/kainate stimulation causing long-term neurodegeneration.

In a process similar to depression, there is increasing evidence of the roles played by inflammation and immunotoxicity in the pathogenesis of schizophrenia. Quinolinic acid, from the kynurenine pathway, is a potent pro-inflammatory agent and also an NMDA receptor agonist. In post-mortem studies, immunoexcitotoxicity is accompanied by an increased number of T-lymphocytes in brain tissue [125]. The inflammatory mediator TNF-alpha stimulates microglial glutamate release, inhibits GLAST and GLT-1 reuptake transporters, and increases astrocytic glutamate synthesis. TNF-alpha also upregulates AMPA receptors causing Ca^2+^ influx and the conversion of microglia into pro-inflammatory phenotypes. Increased glutamate triggers mGluRs on microglia to release TNF-alpha. Collectively, the effects of TNF-alpha cause excitotoxicity and increased microglial inflammation [5]. Of the two processes, the pathway responsible for the subsequent neurone destruction is unclear. Whether microglial activation and mediator release is present in all cases of schizophrenia, or a subset, is also unclear. Notably, some current antipsychotic treatments also suppress inflammatory cytokines and possibly microglial activation, although the beneficial or detrimental effects of this are unknown [126]. 

There is no doubt that immunoexcitotoxicity occurs in the initial pathogenesis of schizophrenia, possibly starting as early as during pregnancy [127]. Toxicity at this stage causes both disrupted neurodevelopment and neurodegeneration to contribute to the course of the disease, and the pathogenesis shares features in common with neurodevelopmental disorders. The synaptic responses in the presence of immune mediators, in adult people with schizophrenia, are heightened compared to healthy individuals, suggesting previous priming events by immune stimulation, such as infection in utero or the neonatal period or repeated excessive inflammatory responses to stimuli during childhood [128]. Similar priming and triggering events are seen in the development of autism [129,130]. It has been shown clearly that prenatal infections in the mother can cause postnatal schizophrenia, although it is the stimulated inflammatory response, not the infectious agent, that appears to be responsible [131].

Microglial cells in the CNS are primed by infiltrating peripheral inflammatory mediators. Cell priming involves proliferation and increased enzymatic activity, generating excitotoxins such as quinolinic acid and inflammatory mediators. Similar to other immune-priming processes, the activation and release of mediators by microglia do not occur until a subsequent immune challenge presents, often years later. Activation involving toxin and mediator release causes dendritic and axonal destruction, especially of the frontal cortex and temporal areas [132]. The process probably occurs in the years prior to overt symptoms appearing, as there is clear evidence of long-standing destruction of brain networks and neuronal loss by the time of diagnosis [133]. Chronic pro-inflammatory states certainly play a role, as people with confirmed schizophrenia demonstrate higher levels of brain and circulating pro-inflammatory mediators, which appear to indicate a continuous, persistent pro-inflammatory excitatory process [134].

As the immunoexcitotoxicity process progresses, the continued overstimulation of glutamate receptors eventually causes neuronal death, with further microglial activation causing the release of inflammatory mediators, which perpetuates the process. Individual neuronal death leads to an eventual reduction in regional glutamate levels, especially in the prefrontal cortex, as seen in proton MRS studies. This manifests clinically as a decline in memory, cognition, and general functioning over time. Recent research has demonstrated that memory recall in the visual neocortex is strongly dependent on hippocampal glutamate excitatory levels and may explain why memory is disrupted in numerous neuropsychiatric disorders [135]. 

Research into the longitudinal course of schizophrenia is complicated by the pro-inflammatory lifestyles of many patients including smoking, poor diet, and sedentariness [136,137]. The hypotheses and evidence surrounding glutamate mechanisms therefore offer new therapeutic biomarkers and targets for earlier diagnosis and safer treatments and is an area of intense research. 

Immune profiles in plasma show potential for identifying those patients at risk of worse outcomes. Studies have attempted to correlate high levels of specific biomarkers such as C-reactive protein (CRP), IL-6, IFN-γ, TNF-α, and genetic polymorphisms of cytokines, and match them with clinical subgroups such as prodromal, first-episode psychosis, chronic psychosis, and negative symptoms, with the aim of developing targeted treatment approaches and more personalized medicine [138]. For example, serum levels of the chemokine MIP-3α during the first 3 months of antipsychotic treatment and the score in negative psychotic symptoms 3 months after the initiation of antipsychotic medication acted as predictors of the initial time to remission of positive psychotic symptoms [139]. Time to remission correlates with worse long-term functional outcomes. Immune targets also offer a potential treatment approach with, for example, immunomodulators such as non-steroidal anti-inflammatory drugs (NSAIDS), minocycline, or anti-monoclonal treatments currently in use for autoimmune disease (e.g., anti-TNF therapeutics). A systematic review showed symptom improvement with these groups of drugs, but not for anti-inflammatory drugs such as oestrogen, statins, or glitazones [140]. In a separate study, probiotics have been shown to modulate inflammatory profiles in schizophrenia, demonstrating the effect of the microbiome, as discussed below [141].

## 5. Neurodevelopmental Disorders

Neurodevelopmental disorders (NDD) are a heterogeneous group of conditions that affect the development of the CNS in childhood and adolescence, although there is a considerable overlap of symptoms within the group. Disorders such as autism spectrum disorder (ASD) and attention deficit hyperactivity disorder (ADHD), previously considered in childhood, can be lifelong [142]. Abnormal brain function may cause difficulties with social behaviour, learning ability, self-control, and memory, often on a spectrum of severity. The global nature of domains potentially affected during development has led to continued revisions of definitions and classifications. In part, this is due to the difficulty of defining normal/abnormal thresholds of function. As a result of this and the uncertainty of the underlying pathogenesis, symptoms are clustered into trait-based clinical classification, whereby functional difficulties are prioritised over pathological lesions, particularly where a person may fall across the boundaries of several diagnoses. Etiological factors, including genetic, infectious, immune, and traumatic, incurred during the perinatal period are strongly associated with NDD, but the precise pathogenesis and mechanisms are still uncertain [143]. Monogenic neurodevelopmental disorders such as Fragile X syndrome (FXS) therefore became the focus of research in finding a model of the underlying pathophysiology. Research in FXS strongly indicates glutamate receptor dysfunction, but phase 2 and 3 trials of plausible drugs have failed to show efficacy. This demonstrates the current extent of the knowledge gaps in FXS and other neurodevelopmental disorders [144]. 

Research into the initiating processes of abnormal neurodevelopment is often necessarily centered around animal models, genomics, and post-mortem evidence, as CNS imaging during the perinatal period, at the time of pathogenesis, is not feasible. Interconnecting neurotransmitter systems mature during the third trimester of pregnancy, postnatal period, and early infancy, and disruption at this time causes both local damage as well as disruption of the developing structure [145].

With later stages of child development, from 2–6 years and onwards, functional MR can interrogate brain biochemistry in vivo for markers such as *N*-acetylaspartate (NAA)/Cr, choline (Cho)/Cr, myoinositol (MI)/Cr, and the glutamine and glutamate complex (Glx)/Cr, using creatine as a reference standard. Metabolic dysfunction is seen in the cerebellum and thalamus in children with ASD, with some indices relating to individual symptoms [146]. In autism, serotonin function is seen to be reduced, although no definitive mechanism has been found to date [147]. Serotonin is involved in neuronal growth and plasticity. Clinically, the reduced activity causes deficits in social interaction, language, and repetitive patterns of behaviour. Imaging of affected young adults shows increased cortical volumes and macroscopic anatomical changes [143]. Serotoninergic activity is driven by competing forces of glutamatergic excitation and GABA inhibition. Indeed, from a glutaminergic perspective, schizophrenia and autism share many features of disrupted synaptic plasticity affecting learning and memory [148,149]. Individual MRS studies of ASD are far from conclusive, with different studies reporting both increased and decreased glutaminergic activity [150,151]. To our knowledge, there are no reported meta-analyses to date examining MRS changes in ASD; reviews have collated findings without determining overall effect sizes or heterogeneity for any given ASD cohort [152]. More recent studies have combined (1)H-MRS with fMRI to study glutamine/glutamate and the excitatory/inhibitory (E-I) balance in vivo, correlating activity with regional anatomy and finding increased glutamate excitation but reduced interregional connectivity [153]. Others have correlated glutaminergic function with clinical features, such as sensory hyper-reactivity [154].

### 5.1. Obsessive Compulsive Disorder

Obsessive compulsive disorder (OCD) is characterized by persistent intrusive thoughts (obsessions) and repetitive behaviours (compulsions). OCD has a prevalence rate of 1–3% in the general population and is a common cause of illness-related disability [155]. There are known genetic and environmental factors influencing pathogenesis and hypotheses. Since the early 2000s, the hypothesised mechanism suggests increased glutamatergic excitation, with or without reduced GABA inhibition [156]. MRS in OCD reveals glutamatergic hyperactivity in the orbitofrontal cortex [157]. This may be a result of glutamate transporter dysfunction as this is demonstrated in animal models of OCD [158].

### 5.2. Neurodevelopmental Genetic Associations

Across neurodevelopmental disorders, heritable defects are being investigated by associating anatomical abnormalities on MRI with single-nucleotide polymorphisms (SNP) from glutamatergic candidate genes. Brain regions showing strong associations with SNPs are the anterior cingulate cortex, thalamus, and orbitofrontal cortex [159]. These genes encode principal components of the molecular machinery that connects pre- and post-synaptic neurones, facilitates glutamatergic transmission, controls synaptic plasticity, and empowers intersecting neural circuits to process and refine information. For ADHD, genome-wide analyses for risk genes reveal glutamate receptors (GRM5) and mediators of intercellular signalling [160]. Recent advances in high-throughput DNA sequencing have revealed mutations of genes coding for glutamate receptors. Notably, gene mutations of iGluRs are strongly associated with intellectual disability and autism spectrum disorders. In contrast, mutations of metabotropic GluRs, with a role in modulating neural transmission, are preferentially associated with psychiatric disorders [161]. Combined functional, anatomical, and genetic research aims to identify causative factors and targets in a similar process to other glutamatergic-related conditions. 

## 6. Neurodegenerative Conditions and Glutamate

Neurodegenerative diseases remain some of the most challenging healthcare conditions to treat. In many cases, underlying disease mechanisms are poorly understood, and there is currently no therapy that halts disease progression [162]. Numerous neuroprotective drugs have failed to show any significant clinical benefit in trials despite convincing rationale. This makes the need for further determining mechanisms and identifying new targets more imperative [163]. Genetic diseases such as the autosomal recessive Niemann–Pick disease, with known biochemical defects of myelin sheath production and maintenance, provide insights into degenerative processes and are suggestive of glutamate involvement [164]. Excitotoxicity by glutamatergic overstimulation is becoming recognised as a central feature of many neurodegenerative diseases, including Alzheimer’s disease (AD), Parkinson’s disease, and Huntington’s disease [165,166,167]. The glutamatergic roles in memory, learning, and neuroplasticity are reflected in the symptomatology of these conditions, which display these deficits. Characteristic pathological features of a neurodegenerative process are impaired astrocytic ability to uptake and respond to glutamate and the associated rise in extracellular glutamate causing neuronal overstimulation, oxidative stress, toxicity, and mitophagy-mediated cell death [168,169]. In addition to increased glutamate levels, abnormalities of glutamate receptors on glia and neurones are also clearly involved. In Alzheimer’s disease, mGluRs on microglial cells are centrally involved in neuroinflammation and neurotoxicity. Recent studies show the role of microglial cells in the progression of AD through alterations in Ca^2+^ signalling, amyloid precursor processing, and beta-amyloid generation to produce the characteristic senile plaques [170]. There is also evidence of decreases in glutamate reuptake mechanisms and increased glutamate release [171]. One hypothesis proposes the cellular senescence theory of astrocytes, featuring reduced cell proliferation, a pro-inflammatory phenotype, and downregulation of glutamate transporters, collectively increasing extracellular glutamate [172].

Other hypotheses cite peripheral pro-inflammatory states triggering central glutamatergic dysfunction and degeneration. For example, both long-standing type 2 diabetes and chronic periodontitis are associated with Alzheimer’s disease [105,173]. Although a mechanism or agent has never been established in periodontitis, inflammatory processes are suspected. Theoretically, drugs that reduce excitotoxicity may help slow the process, and glutamate modulators, such as Memantine, an NMDA receptor antagonist, are already in routine clinical use for Alzheimer’s disease [174]. Drugs such as Riluzole, used to treat amyotrophic lateral sclerosis, reduce the release of glutamate, whilst other diverse treatments that block excitotoxicity, including hypothermia, fingolimod, minocycline, ketogenic diets, and glutamate dialysis, are being evaluated [163,175,176].

In summary, the close and complex system of interrelations between glutamate neurones and astroglia suggests that even simple dysfunctions or damage from external agents ripple out to cause complex and unpredictable consequences over time, making the identification of the initial processes prior to neuronal death extremely difficult. Hence, the current understanding and progress in drug development for neurodegenerative diseases remain in the early stages. 

## 7. Epilepsy 

Epilepsy is characterized by seizures, although the definition is problematic as not all seizures are due to epilepsy and diagnostic EEG testing can be inconsistent [177]. As glutamate is the main excitatory neurotransmitter in the CNS, it is accepted that glutamatergic hyperexcitation is causative in initiating seizures, with NDMA and AMPA receptors being implicated. Glutamate receptor antagonists inhibit seizures in animal models and are therefore the focus of glutamatergic-based treatment research for epilepsy [178].

## 8. Brain Injury

Traumatic brain injury (TBI), from external trauma, or brain injury from stroke, can also result in secondary neural damage from injury-induced glutamate release and excitotoxicity. Although incompletely understood, inflammatory and repair processes including activation of the kynurenine pathway (with inflammatory activation of astrocytes and microglia) lead to increased production of free radicals and excitotoxins, including quinolinic acid, resulting in neuronal dysfunction and apoptosis [179]. Environmental, lifestyle, or psychological stresses on the person after the injury can impact the ability of regulatory genes to contain this neuroinflammation. This can lead to long-term cognitive impairment, disproportionate to the original injury, and may reflect a continuing neurodegenerative process. Indeed, TBI is a risk factor for developing Alzheimer’s disease [180]. The clinical measures of cognitive impairment in TBI are seen to correlate with quinolinic acid levels in cerebrospinal fluid in those patients with poorer outcomes [181]. To help identify TBI patients at risk of post-traumatic neurodegeneration, MRS has shown utility in those patients with negative routine CT and/or MRI scans, in the subacute post-injury phase (<90 days). A meta-analysis of MRS studies using *N*-acetyl-aspartate (NAA) as a marker of neuronal integrity and glutamate (Glu) as a marker of disturbed brain metabolism showed a reduced NAA/Glu ratio in TBI patients, primarily in the frontal lobes, indicating a possible use as a diagnostic tool [182].

## 9. Post-Traumatic Stress Disorder (PTSD)

PTSD is a debilitating mental illness, involving distressing thoughts and feelings of a recurring nature, persisting beyond one month after a traumatic event. The exaggerated responses to trauma-related cues are distressing to the person and lead to behavioural changes of cue-avoidance or dysfunctional coping strategies. Physiologically, there is dysregulation of the normal stress response, due to structural and functional neuronal changes and dysregulation of the HPA axis and adrenal catecholamines. The close relationship between glutamatergic activity and the HPA and sympathetic–adrenal axes and the increasing evidence of abnormal glutamatergic responses to stress add to previous evidence of serotoninergic and GABA system dysfunction in PTSD [183]. Hippocampal atrophy results from stress-induced glutamate excitotoxicity and is implicated in the recurrent re-experiencing of symptoms and other memory impairments. Lower hippocampus levels of NAA and increased glutamate on MRS do correlate with the degree of flashback re-experiencing in PTSD patients [184].

This highlights glutamate as a potential new target for therapeutic development with drugs such as ketamine, which is currently under clinical evaluation [185,186]. Currently, the most effective serotonin-based drug treatment, Sertraline, only achieves a 60% response rate [187]. Notably, TBI seems to damage brain structures that are predisposed to the development of PTSD, and in practice, there is a strong correlation between the two conditions, in both clinical and animal studies [188].

## 10. Other Disorders 

Numerous other diverse neuropsychiatric conditions exist where research is challenging existing hypotheses and revealing glutamatergic involvement:

(1) Binge eating disorder affects 3.5% of the population and, together with similar eating disorders, causes significant disability. Symptom overlaps occur with substance use disorders, and initial research indicates the involvement of GABA and glutamate modulation pathways and beneficial responses to glutamatergic modulators [189].
(2) Anorexia nervosa is the most serious of the eating disorders, associated with high morbidity and mortality. However, the pathogenesis is poorly understood and there are no established pharmaceutical treatments. High field strength MRS (7T) shows reduced glutamate in the anterior cingulate cortex, occipital cortex, and putamen [190].
(3) Personality disorders (PD) are a diverse group of mental disorders associated with dysfunction of social interactions and suicidal risk. Different subtypes have very different clinical presentations but all share difficulty with rigidity of thinking, social functioning, and behaviour, deriving from how the person perceives themselves and their relationships with others. Previously thought to be a “psychological” disorder with no organic basis, increasing evidence highlights abnormalities of prefrontal cortex glutamate activity. MRS studies show significant differences in NAA and Glu in the dorsolateral prefrontal cortex (DLPFC) between PD and healthy controls and across PD subtypes [191]. Interestingly, studies in PD have shown increased central pain suppression, pain adaptation, and dissociation from pain, especially in women and the borderline personality disorder subtype. Although the mechanisms are unclear, this hyposensitivity may underlie the high incidence of self-harming behaviour associated with this condition [192].

## 11. Effects of Gut Microbiota on CNS Disorders and Glutamate Dysfunction

In tandem with research on the integral role of the gut microbiome in general health, there is increasing evidence of the gut microbiome’s involvement in CNS diseases, as both a cause and mitigator. Next generation sequencing technology has enabled detailed mapping of the microbiome constituent species. Evidence indicates beyond doubt that, collectively, microbiota products play similar roles in brain homeostasis as in other organs, including energy metabolism, tissue growth, micronutrient synthesis, immune function, and nervous system development. In brain development studies, germ-free mice lacking gut microbiota are seen to have a heightened stress response in adulthood, which can be reversed by microbial colonisation [193].

The extent of transport from the gut to the brain of metabolic, immune, and growth factors, neurotransmitters, and substrates is considerable and is still incompletely described. Collectively, this is termed the gut–brain axis. Of the multitude of peptides and mediators, the main classes are short-chain fatty acids, bile acids, tryptophan catabolites for serotonin and the kynurenine pathways, glutamate, and GABA. Dysfunction of the gut–brain axis has bidirectional effects: Microbiota imbalance causes CNS changes in BDNF, serotonin receptors, and synaptic plasticity, whilst individuals with psychiatric illness have reduced diversity of healthy microbes compared to healthy individuals [194,195]. There are undoubtedly innumerable other gut-derived molecules, so far undefined, which modulate aspects of brain development, activity, and repair. As many of these agents are bacterial species-specific, the proportion of different microbe populations in the gut affects the profile of signals travelling to the brain and causes shifts in CNS metabolic and inflammatory activity and neurotransmission modulation. Glutamatergic activity in the brain may be directly affected by the gut–brain axis. A recent study in mice used mass spectrometry of phosphorylation sites in hippocampus tissue to examine the gut–brain axis effects on the brain of depressive and control-type microbiota profiles [196]. The phosphorylation dysregulations were consistently associated with glutamatergic neurotransmitter disturbances. 

A healthy microbiome appears to be necessary in maintaining CNS glutamatergic integrity via modulatory pathways such as the kynurenine pathways or short-chain fatty acids. Brain BDNF, which is required for maintaining NMDA receptors, is reduced in germ-free mice. Reduced NMDA activity and hence lower GABA inhibition on glutamate leads to synaptic dysfunction and reduced cognition. Manipulating gut microbiota with prebiotics, probiotics, and antimicrobial drugs appears to influence symptom severity in several neuropsychiatric conditions [197].

Evidence of non-intuitive links between microbiota and CNS diseases is continually emerging. A systematic review that included 17 articles showed that gut dysbiosis predicts the development of neurological or neuropsychiatric diseases such as cognitive impairment, Alzheimer’s disease, Parkinson’s disease, and depression [198]. In addition, it was found that different psychobiotics, mainly dietary fibres and probiotics of the Lactobacillus family, improved different cognitive functions such as cognitive performance and induced a reduced glucocorticoid response. Diseases such as ASD, multiple sclerosis, Parkinson’s disease, schizophrenia, and Alzheimer’s disease are associated with distinct microbiota changes corresponding to patterns of CNS inflammation or excitotoxicity [199,200,201,202]. In people with depression, the microbiota was found to contain fewer Faecalibacterium and Coprococcus than healthy controls [203]. Establishing causality beyond correlation in this bidirectional gut–brain axis has proved extremely problematic, but early research indicates the potential for numerous novel targets and agents and is the subject of intense research. 

Despite the increasing complexity of the taxonomy of the microbiome and challenges of establishing mechanisms of CNS disease, empirical evidence for the modulation of illness by diet and probiotics has existed for centuries. In a systematic review of existing trials of probiotics to treat depression, the aggregated depression symptom score was significantly reduced with probiotics. Notably, the effect size was larger and more significant in people under 60, although the reason for this is unclear (MD = −0.43, 95% CI (−0.72, −0.13), *p* = 0.005) [204].

In a different form of microbiome modulation, faecal microbiota transplant (FMT) research originated from 4th Century China, using faecal slurry for treating diarrhoea [205]. Over the centuries, FMT has been used sporadically in different contexts. Recently, FMT research has demonstrated significant effects on several neuropsychiatric diseases. There is strong systematic review evidence of FMT both alleviating depressive symptoms in patients and inducing depressive symptoms in healthy FMT recipients [206]. The transmission effect even applies, with FMT from patients with schizophrenia causing schizophrenia-like behavioural abnormalities in recipient mice [207]. Therapeutic FMT is currently being researched in autism, MS, and Parkinson’s disease [208,209,210]. A variation of this technique is to culture faecal colonies, then lyophilize them (freeze-dry) and provide them as an oral preparation. This is being considered in MDD [211].

## 12. Glutamatergic Function and Exercise 

Exercise profoundly affects neuroplasticity and neurogenesis and therefore benefits cognition. Physical activity in humans especially promotes hippocampus-dependent memory and prefrontal cortex executive function, two brain regions where glutamatergic activity is most intense. Exercise also has long-term benefits in maintaining cortical volume over the lifetime. Initial CNS responses to exercise are rapid, and neurotransmitter levels and blood flow increase. Growth factors such as BDNF then appear, followed by neurogenesis in the hippocampus [212]. Many of these effects are glutamate mediated as, in addition to its role as a neurotransmitter, glutamate also regulates neurogenesis, neurite outgrowth, synaptogenesis, and neuron survival [213]. It is these effects by which exercise can also exert a neuroprotective preconditioning effect, protecting the brain from ischaemia [214]. Exercise has long been associated with significant benefits in neuropsychiatric disease, but the mechanisms are still unclear. Many of the neuroinflammatory, excitotoxicity, and neurodegenerative mechanisms of glutamate dysfunction, discussed above, are ameliorated by exercise. Exercise reduces glutamatergic activity and increases growth factors, even in abnormal brain tissue where these functions have been previously impaired. Whilst there is no doubt that exercise benefits depression, there is less certainty about the role of exercise in halting or even reversing established neurodegenerative disease [215]. A systematic review showed that exercise improved cognitive functioning in schizophrenia and stereotypic behaviours, as well as social-emotional functioning, cognition, and attention in autism [216,217]. For established Alzheimer’s disease, systematic reviews of clinical studies have been inconclusive, but reviews of exercise in prevention show strong effect sizes of up to 28% [218,219,220].

## 13. Conclusions

Glutamate neurones, surrounding astroglial cells and inhibitory GABA neurones, collectively form a functional unit of synaptic excitation/inhibition that is replicated throughout many regions of the CNS. Evidence from clinical imaging and longitudinal studies, animal models, and in vitro cell work suggests causal relationships between altered glutaminergic excitation at synapses and clinical symptoms of depression and many other neuropsychiatric conditions. In the case of depression, the provoking agents and targets, leading to overstimulation in the hippocampus, offer a new paradigm of targets for potential therapeutics. The evidence of similar glutamatergic excitotoxicity in other neuropsychiatric diseases suggests a common theme of an excitatory/inhibitory imbalance and neuroinflammation, with eventual neuronal and astroglial atrophy, with the anatomical sites and time affected determining the symptomatology. The effectiveness of glutamatergic-modulating drugs in previously diverse conditions such as autism and Alzheimer’s disease provides further evidence of almost universal glutamate involvement in neuropsychiatric illnesses. However, many plausible drugs still fail in trials, indicating the incomplete current state of knowledge and the need for further research.

## Data Availability

Not applicable.
